# Acute Intermittent Dyspnœa

**Published:** 1829-04-01

**Authors:** 


### XXXIV.

### HOPITAL DE BEAUCAIRE.

### ACUTE INTERMITTENT DYSPNŒA.

The following very interesting case, exemplifying one of the anomalous forms which ague assumes, is recorded by Dr. Bland, senior physician to the Beaucaire hospital.

*Case.* On the 19th September, a little girl of 5 years of age, who had experienced a fright some days previously, was seized with difficulty of breathing, cough, and mucous expectoration, unaccompanied by fever or any thoracic pain. This continued till the night of the 23d, when the oppression was unusually great; and, from this period, the complaint assumed the intermittent, or at least the remittent character. Every morning at 2 or 3 o'clock the paroxysm of dyspnœa came on, and continued for several hours. The cough was then almost incessant; induced vomiting; and the child was threatened with suffocation. On the morning of the 9th October, the paroxysm came on at 5 o'clock—was more severe than any preceding attack, and was in full force at 10 o'clock, when the patient was carried to the hospital. The face was now pale, the lips livid, the eyes without expression, the respirations 60 in the minute, and carried on with great difficulty. On applying the stethoscope, the *râle sibilant*, mixed with the râle muqueux, was heard. Vomiting was occasionally induced by the cough. In the midst of all this commotion of the respiratory system, the other functions remained undisturbed. The intellects were clear, the tongue moist, no thirst, the pulse regular and 75, the temperature of the skin natural. These apyrectic phenomena induced Dr. B. to prescribe a mixture of antispasmodics with laudanum, by which the paroxysm was soon brought to a termination. In the evening there was a complete intermission. The sulphate of quinine was then immediately exhibited, and not a single paroxysm returned.

Dr. B. asks what was the nature of the above complaint? and comes to the conclusion that it was one of those mysterious maladies which, for want of a better term, we must designate "A NERVOUS AFFECTION." He thinks the seat of the complaint (whatever may have been the cause) was in the pneumo-gastric nerves, and that the effect was a spasm on the orifices of the air-cells, and constrictor muscles of the larynx. Hence the dyspnœa, cough, and the other phenomena detailed above To this explanation we do not object, as far as it goes. But we have no hesitation in considering the malady in question as *caused* by the same invisible agent or agents which give birth to the whole class of intermittent affections; and, in short, that it was as much one of these last as the ague which shakes the whole system to its centre, or the supra-orbital neuralgia which affects only a single nerve. Dr. Ley has recently given an instance of intermittent cough, in the person of a lady, and which was mistaken for some time, till the periodicity was detected, when the quinine soon put a period to the complaint. If practitioners will be on their guard, and keep a keen eye on their patients, they will very frequently find periodicity when they think they have continuity to deal with. This is not a matter of mere curiosity—it will tell strongly on the results of their practice.

---

### XXXV.

### GLASGOW ROYAL INFIRMARY.

"SINGULAR CASE OF EMPHYSEMA."*

